# P05.16. Subjects’ experiences of a bi-weekly yoga and Ayurveda-based weight loss protocol in overweight and obese women

**DOI:** 10.1186/1472-6882-12-S1-P376

**Published:** 2012-06-12

**Authors:** T Braun, L Conboy

**Affiliations:** 1 Kripalu Center for Yoga and Health, Lenox, USA; 2 Harvard Medical School Osher Research Center, Boston, USA

## Purpose

Yoga is one of the most common forms of complementary and alternative therapies utilized for weight loss. This mixed methods study included quantitative surveys and interviews of participants of a 10-week, bi-weekly yoga and Ayurveda-based weight loss program designed for overweight and obese women. The program involved training in yoga poses, breathing, and philosophy as well as eating mindfully, cultivating relaxation, and compassion.

## Methods

This analysis focuses on the subject interviews which occurred both pre and post program and covered the topics of: (1) hopes and found benefits of the program, (2) anticipated challenges and lessons learned, (3) subjects’ relationships with food pre and post program. Analysis was directed by a grounded theory approach. The interview notes were double coded independently by the two authors. Any disagreements in coding were addressed in discussion. Coding consisted of searching for sought themes and emergent themes in each transcript as well as condensing responses to each topic across the sample.

## Results

Thirty-seven overweight and obese participants were enrolled (mean BMI of 34.1; SD +/-6.1) with mean age of 48.3 years. Most were yoga naïve and had tried weight loss programs in the past. The qualitative findings support the trial’s quantitative findings of statistically significant improvements baseline to post program in BMI (p<0.05), and a number of psychosocial variables. Pre-program, subjects reported that they were drawn to the yoga-based program to achieve greater clarity, structure, and security in their lives in addition to losing weight. Post program, subjects reported greater sense of security, self-confidence, self-compassion, and insight into their behavioral motivations. Useful program tools mentioned are: breath work, mindful eating, focus on self-care, relaxation.

## Conclusion

Our data suggests that participation in a 10-week yoga and Ayurveda-based weight loss program is associated with improvements in reported psychosocial health immediately following program completion.

